# Large-scale acoustic-driven neuronal patterning and directed outgrowth

**DOI:** 10.1038/s41598-020-60748-2

**Published:** 2020-03-18

**Authors:** Sharon Cohen, Haim Sazan, Avraham Kenigsberg, Hadas Schori, Silvia Piperno, Hagay Shpaisman, Orit Shefi

**Affiliations:** 1 Faculty of Engineering, Bar-Ilan University, Ramat Gan, Israel; 2 Bar-Ilan Institute of Nanotechnologies and Advanced Materials, Bar-Ilan University, Ramat Gan, Israel; 3 Gonda Multidisciplinary Brain Research Center, Bar-Ilan University, Ramat Gan, Israel; 4 Department of Chemistry, Bar-Ilan University, Ramat Gan, 5290002 Israel

**Keywords:** Biophysical methods, Neurology

## Abstract

Acoustic manipulation is an emerging non-invasive method enabling precise spatial control of cells in their native environment. Applying this method for organizing neurons is invaluable for neural tissue engineering applications. Here, we used surface and bulk standing acoustic waves for large-scale patterning of Dorsal Root Ganglia neurons and PC12 cells forming neuronal cluster networks, organized biomimetically. We showed that by changing parameters such as voltage intensity or cell concentration we were able to affect cluster properties. We examined the effects of acoustic arrangement on cells atop 3D hydrogels for up to 6 days and showed that assembled cells spontaneously grew branches in a directed manner towards adjacent clusters, infiltrating the matrix. These findings have great relevance for tissue engineering applications as well as for mimicking architectures and properties of native tissues.

## Introduction

The architecture of the brain and the nervous system is very complex, yet it is well organized^1,2^. High-level architectures can be found in the spinal cord^3,4^, retina^5,6^, six-layered cortex^7^, hippocampus^8,9^, cerebellum^10^, and many other neuronal sub-regions. Between these organized sub-regions, specific neuronal networks, consisting of myriad neurons, work together to transfer and process information efficiently and to perform specific tasks that later are translated into various behaviors^2,11,12^. Hence, the ability to artificially organize and pattern neurons at specific spatial positions in order to mimic different architectures and properties of neural networks is of great importance. In various fields in neuroscience research such as neural tissue engineering and regenerative medicine^13–16^, brain-machine interface^17–19^, developmental neuroscience^20^, and neuropharmacology^21,22^ such controlling ability may be crucial.

Indeed, in neural tissue engineering, much effort has been devoted to artificially mimic the highly organized architectures and properties of native tissues. Tang-Schomer and colleagues engineered functional brain-like cortical tissue by assembling donut-shaped layers. Their work offers a model with physiologically relevant features for assessing different brain disorders^23^. Qian and colleagues have generated forebrain-, midbrain-, and hypothalamic-specific organoids from human-induced pluripotent stem cells, which were able to recapitulate the key features of these brain regions^24^. Current methods for arranging specific cells include selective surface modifications^25,26^, direct bioprinting^27,28^, specific micromolding^29,30^, and the use of different forces such as optical^31^, magnetic^32^, and fluidic forces^33^. Although these methods allow successful cell patterning, each technique has its own limitations. For example, bioprinting is costly and time consuming because it is applied sequentially, and its implementation *in vivo* is limited. In addition, parameters such as shear stress on cells, the material’s viscosity, and different droplet sizes can dramatically affect the final result^34^. Magnetic^35,36^ manipulations require introducing non-organic material or other labeling materials that may affect typical cell functioning^37,38^. Optical manipulations are used mainly for single-cell trapping and require high-intensity energy levels. Finally, surface modifications and micromolding are inflexible, requiring a pre-fabrication step.

Acoustic manipulation is an emerging remote, non-invasive, and bio-compatible method^39–41^. In its simplest form, standing acoustic waves are generated when two waves with the same frequency travel in opposite directions. Suspended particles experience hydrodynamic forces, which drive them to specific locations. Particles denser and less compressible than the surrounding medium (thus having what is termed a positive acoustic contrast factor)^42^ will be directed towards the nodal areas of the standing acoustic wave. This method was used to form various microstructures^43^ and to study particle-particle interactions and coalescence phenomena^44^. Since cells usually have a positive acoustic contrast factor in their native environment^45^, acoustic manipulation could be applied on cells without any labeling or surface modifications that may harm the cells. Furthermore, the power density (several orders of magnitude lower than the optical counterparts) and the frequency range used is considered safe and the cells maintain their viability^46^. Finally, since this method can be scaled up and performed inside closed systems, one can envision applying this concept *in vivo*.

In the last two decades, researchers have examined the ability of acoustic standing waves to control the spatial distribution of different cells for various applications in a 2D and 3D manner^46–50^. Despite the advantages of using standing acoustic waves for arranging elements, only a few studies have been conducted using this method for organizing neuronal or neuron-like cells^51–54^. Bouyer and co-authors organized multi-layered 3D constructs of neural progenitor cells (NPCs) derived from human embryonic stem cells within a fibrin hydrogel. Recently, Cheng *et al*. have demonstrated a similar patterning method for PC12 neuron-like cells, where they organized the cells within gelatin-hydroxyphenylpropionic acid (Gtn-HPA) hydrogel, using a glass reflector, to achieve 3D shaping^54^. Gesellchen *et al*. organized acoustically Schwann cells as a template for neurons. Brugger and colleagues have demonstrated long-term manipulation for small-scale patterning (230 cells/mm^2^). They applied the acoustic stimulation for up to 11 hr continuously, affecting both the location and growth^53^. During network formation, neurons transmit information over long distances via their axonal trees. Neuronal processes are elongated in response to orchestrated chemical and physical signals from their environment^55–64^. Therefore, network patterning and neuronal clustering influence their interconnections, activity, and growth patterns^65–67^.

Here we used acoustic waves for large-scale neuronal patterning, and for examining the effect on neurite outgrowth directionality. Two populations of cells were examined: the neuron-like PC12 cells and primary Dorsal Root Ganglia (DRG) neurons. PC12 cells are a commonly used, cost effective neuronal model. Using these cells as a model system allowed us to calibrate and find the ideal parameters for the acoustic manipulation. DRG neurons are primary neurons, especially most relevant to neuronal regeneration platform development and demonstrate extensive axonal growth. We applied two types of standing waves: surface acoustic waves (SAWs) and bulk acoustic waves (BAWs), demonstrating two different types of cell patterning. Acoustic pressure fields generated by SAWs arranged the cells in string shape as parallel lines while the acoustic pressure fields generated by BAWs arranged the cells in concentric patterns, both with a controllable number of embedded cells per cluster. Moreover, we could affect the neuronal growth by manipulating the cell arrangement. We have shown that the assembled lines of cells spontaneously grew their branches out of their clusters towards adjacent clusters after several days of growth. This large-scale neuronal patterning and growth manipulation via acoustic waves, which can be applied remotely, opens up new possibilities for complex cell arrangement with great potential for neuroengineering and neurotherapeutic applications post trauma.

## Results and Discussion

### Cell arrangement using standing SAWs

**Figure 1:**
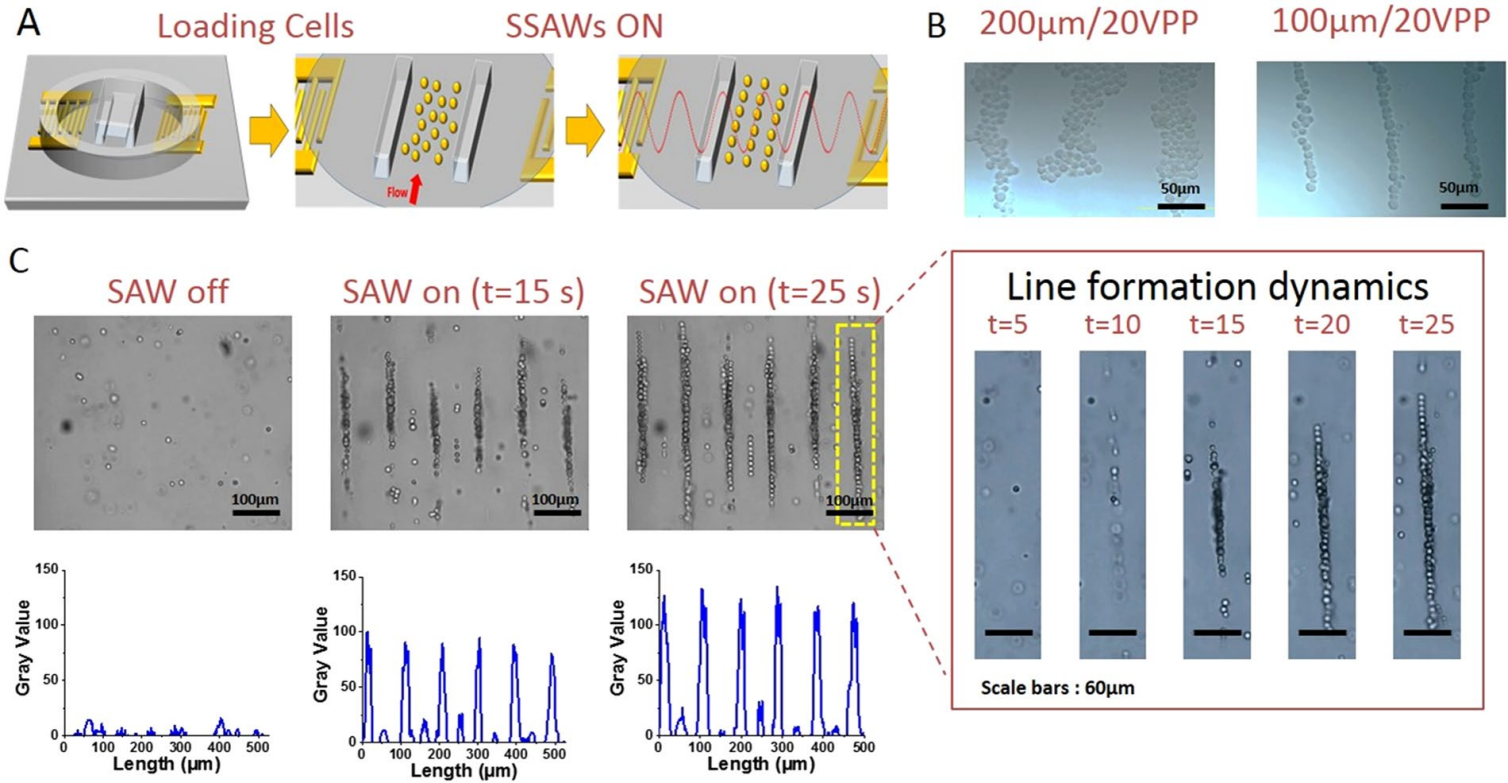
(**A**) Schematic overview of the standing SAW system. Standing SAWs were generated by applying voltage on a series of interdigital transducers (IDTs) that were patterned on a piezoelectric substrate (lithium niobate). The cells are loaded into a 100–200 µm high micro-channel built on the bottom of a glass petri dish. Following the standing SAW activation, the cells align in parallel lines along the pressure nodes. (**B**) Microscope images of patterned cells in channels at different heights, demonstrating the ability to control the thickness of the generated lines. Scale bar = 50 µm. (**C**) Dynamics of the formation of lines for a 200 µm high micro-channel at 10 Vpp. Microscopic images and spatial distribution analysis of cells, demonstrating the fast assembly process. Scale bar = 100 µm. The formation dynamics of one line (yellow dotted rectangle) can be seen in the inset on the right. Scale bar = 60 µm.

To pattern neuronal cells with SAWs, we loaded PC12 cells into a micro-channel (shown in Supplementary Fig. S1) that was placed on top of a pair of interdigital transducers (IDTs), as described in the Experimental section. Standing SAWs formed because of the interference pattern produced by the two identical acoustic waves generated by the two IDTs traveling in opposite directions. The acoustic field generated by the standing SAWs acts to move cells towards defined locations, namely, the pressure nodes, thus forming parallel lines of cells with a distance of half the wavelength of the acoustic wave between adjacent lines^44^ (Supplementary Video SV1 in the Supporting Information). Figure 1A illustrates the experimental process: the setup before cell loading (left panel), after cell loading (middle panel), and after applying the AC signal that generates the SAWs (right panel). Figure 1B presents the patterned cells that were organized as parallel lines after applying a 19.4 MHz radio-frequency signal. We studied how combining different channel heights (ranging from 100 µm to 500 µm) with different applied voltage intensities (ranging from 3 Vpp to 20 Vpp) affects the resulting patterned lines. It can be seen that different channel heights led to different cluster widths. Applying identical voltage intensities (20 Vpp) resulted in much thinner clusters (a width of 1–2 cells) for the 100 µm-high channels compared with the 200 µm-high channels (width of ~10 cells). For 300 µm–500 µm channels, acoustic streaming was generated routinely throughout the entire examined voltage range. This streaming resulted in disassembly of the cells, which started to reassemble as lines.

We found that the applied voltage intensity modulates the line formation dynamics. For the 100 µm and 200 µm height channels, arrangement started slowly at 10 Vpp and became faster as voltage values increased. An example of the assembly procedure’s dynamics for a 200 µm height channel and an intensity of 10 Vpp is shown in Fig. 1C (and Supplementary Video SV1 in the Supporting Information). The spatial distribution of the cells before applying the standing SAWs is homogeneous (Fig. 1C left panel). Once the standing SAWs are applied, the cells respond immediately and move towards the acoustic nodes, thus forming parallel lines (Fig. 1C middle panel). After 25 s the cells cluster as long and dense lines (Fig. 1C right panel and enlarged inset). The increase in the number of cells in the observed area is due to acoustic streaming that directs cells from the surrounding area towards the acoustic nodes.

The standing SAW device shown above is based on a flow of cells through a micro channel open on both sides. This open channel approach is subjected to streaming after turning off the acoustic waves, thus limiting the ability of the cells to stably adhere to the bottom surface. We have found that cells that adhered to the surface, after turning off the SAWs, started to grow, and assumed a normal growth pattern with multiple branches (Supplementary Fig. S2). This observation demonstrates that applying standing SAWs do not compromise the cells’ viability, as was also previously shown for other cell types^49,53,68^.

### Large-scale cell arrangement using standing BAWs

SAW devices are fabricated in-house and can be tailor made for specific cell spacing. SAWs arrangement can reach resolutions of even a few microns, and therefore this method is preferred when there is a need for high-resolution cell patterning. However, these devices require several steps of lithographic processing which makes them costly in terms of time and labor. They are applicable for arrangement of only thin layers of cells. Additionally, they are rather delicate and prone to scratching of the IDTs and breaking of the piezoelectric substrate.

**Figure 2:**
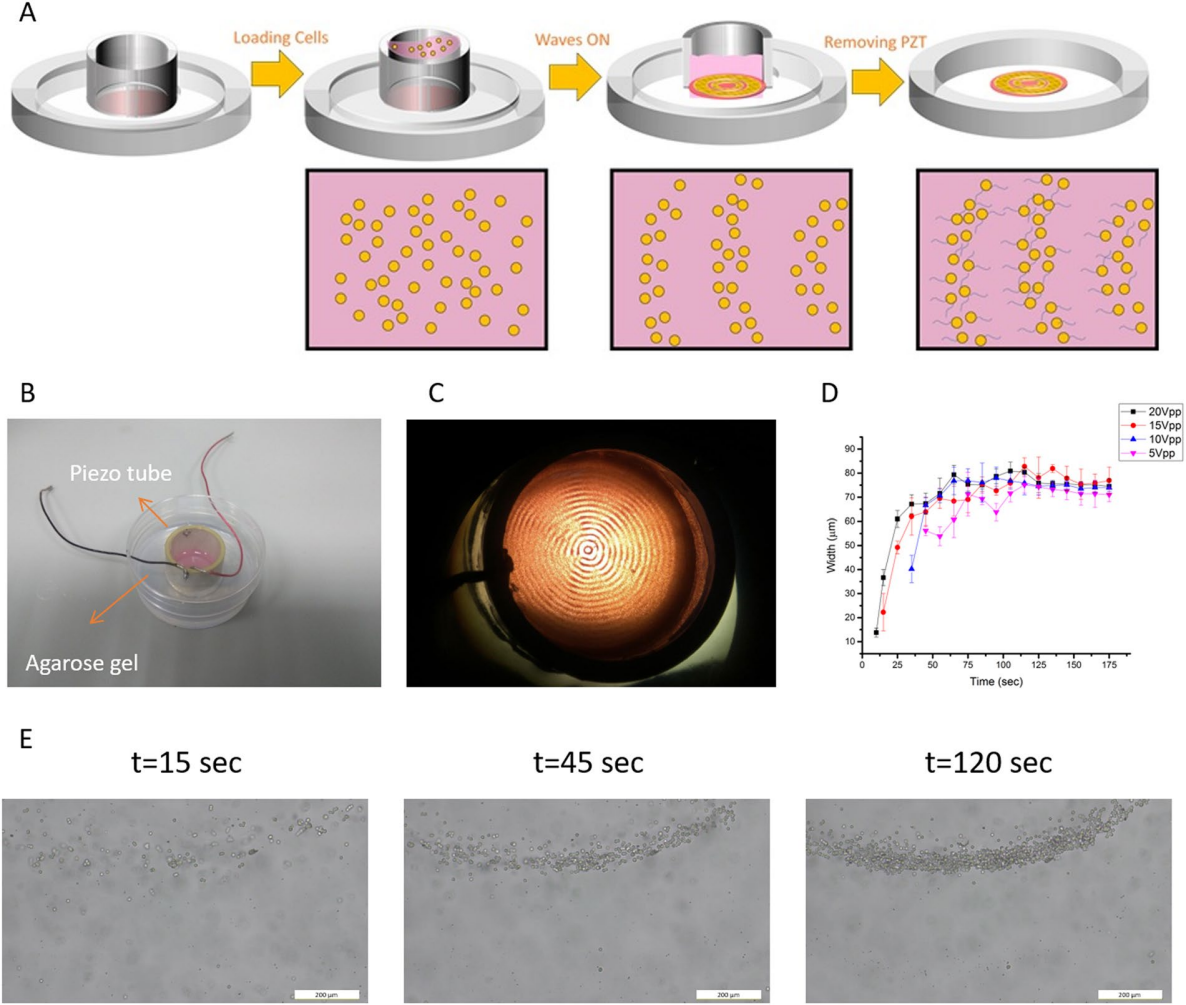
(**A**) Schematic overview of the standing BAW system generated by a radial piezoelectric transducer. Following cell arrangement and attachment to the surface, the piezoelectric transducer was detached, and cells were left to grow branches for several days in an incubator. (**B**) Image of the piezoelectric resonator on a petri dish stabilized with agarose gel used in the experimental setup. (**C**) Image of the directed assembly of particles with 1.14 MHz at 10 Vpp. (**D**) Width (of the fourth ring from the center) as a function of time for various applied voltages using PC12 cells (1 × 10^6^ cells/ml). Each data point represents an average of six individual measurements. (**E**) Dynamics of BAWs cells arrangement at an intensity of 10 Vpp. Microscopic images along time demonstrating the assembly process. Scale bar = 200 µm.

A second acoustic approach we have developed for organizing neuronal cells is based on bulk acoustic waves (BAWs) that allow large scale arrangements in a 3D environment. BAW devices are commercially available, more robust, less expensive, and easier to use compared to SAW devices. In this scheme we used a lead-zirconate-titanate (PZT) piezoelectric radial transducer (22 mm inner diameter) that also served as a reservoir for cells placed at the center of a standard polystyrene petri dish (Fig. 2). To identify the resonance frequency of the transducer, we assembled 2 µm polystyrene (PS) beads in water. We found that applying a frequency of 1.14 MHz led to a rapid formation of well-defined concentric circles of beads (Fig. 2C) with a spacing of about half the applied wavelength following the work of Raeymaekers *et al*.^69^. We examined the effect of different voltage intensities on the widths of the formed circles as a function of time (Supplementary Fig. S3). We found considerable differences between the examined intensity values for activation times less than 5 min (higher intensities led to thinner rings). Since the total number of particles is constant in all cases, thinner rings are associated with denser areas. For activation times longer than 5 min, there was no substantial difference in circle width for various intensities. We also noticed that using 10 Vpp gave the maximal dynamic range (see Supplementary Fig. S3) which allows maximum control over density.

Following the 2 µm polystyrene (PS) beads analysis, we quantitatively analyzed the relation between different voltage intensities (ranging from 5 Vpp to 20 Vpp) to cell arrangement (Fig. 2D). It can be seen that different intensities influenced the onset of the circle formation, with higher voltage intensities leading to faster cell clustering. We note that after nearly 120 sec all cell clusters reached their final width. Figure 2E demonstrates the assembly process dynamics for a 10 Vpp intensity.

Based on the quantitatively analysis of the PS beads and the PC12 cells, we have continued our experiments with an intensity of 10 Vpp. To organize cells, the radial resonator was loaded with PC12 cells, as described schematically in Fig. 2, followed by activation of BAWs (1.14 MHz, 10 Vpp).

**Figure 3:**
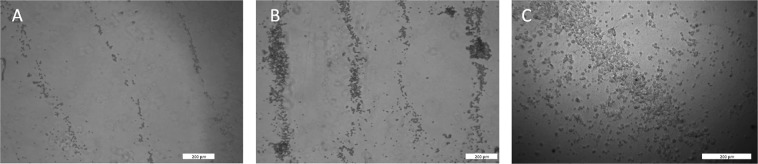
Bright-field microscopic images of acoustically assembled PC12 cells with BAWs at different concentrations: (**A**) 100 K cells/ml, (**B**) 250 K cells/ml, and (**C**) 1 M cells/ml. The images demonstrate that the width of the rings increases with higher cell concentration. Scale bar = 200 µm.

To examine the effect of cell density on circle formation, several concentrations of PC12 cells were loaded into the acoustic reservoir and were subjected to BAWs. The concentrations examined were 1 × 10^5^, 2.5 × 10^5^, and 1 × 10^6^ cells/ml. The cells were subjected to the acoustic field for 10 min (Fig. 3). Clear concentric circles were always observed. As expected, it can be seen that the width of the rings increase with higher cell concentration.

To conclude, the acoustic field generated by the piezoelectric resonator acts by moving cells towards defined locations (nodal areas), thus forming concentric circles. We found that the optimal conditions for the PS beads (2 µm) were also effective for organizing PC12 cells (10–12 µm)^70^, although the size varied. This is in accordance with previous studies that have demonstrated acoustic manipulations of different sizes of beads (from 8 µm to 202 µm) followed by manipulation of cells^52^. The distance between adjacent assemblies was always identical for a given frequency. Our formed concentric patterns resemble natural organization in the nervous system. Specifically, it may mimic brain areas such as the pyramidal layers in CA1 to CA3 in the hippocampus^71,72^ or the Purkinje layer in the cerebral cortex^73^. In the brain, neuronal densities vary across and within cortical areas^74–76^, presumably due to different information-processing demands. Therefore, our findings regarding the ability to control cells density by using different cell concentrations and voltage intensities may allow fitting the assembled pattern to the physical properties of the desired tissue. In future applications this method can be expanded, For example, by using holographic acoustic tweezers^77^, 3D complex brain-like organoids can be formed^78,79^.

### Directed branches’ outgrowth on top of a 3D collagen hydrogel following acoustic manipulation

To study the effect of BAWs on cell growth, two model systems were studied: differentiated PC12 cells and primary dorsal root ganglion (DRG) neurons. We kept the BAWs active for 30 min to allow the cells to settle on top of the gel substrate. Then, the tube was removed and the cells were kept up to a week in the incubator.

**Figure 4:**
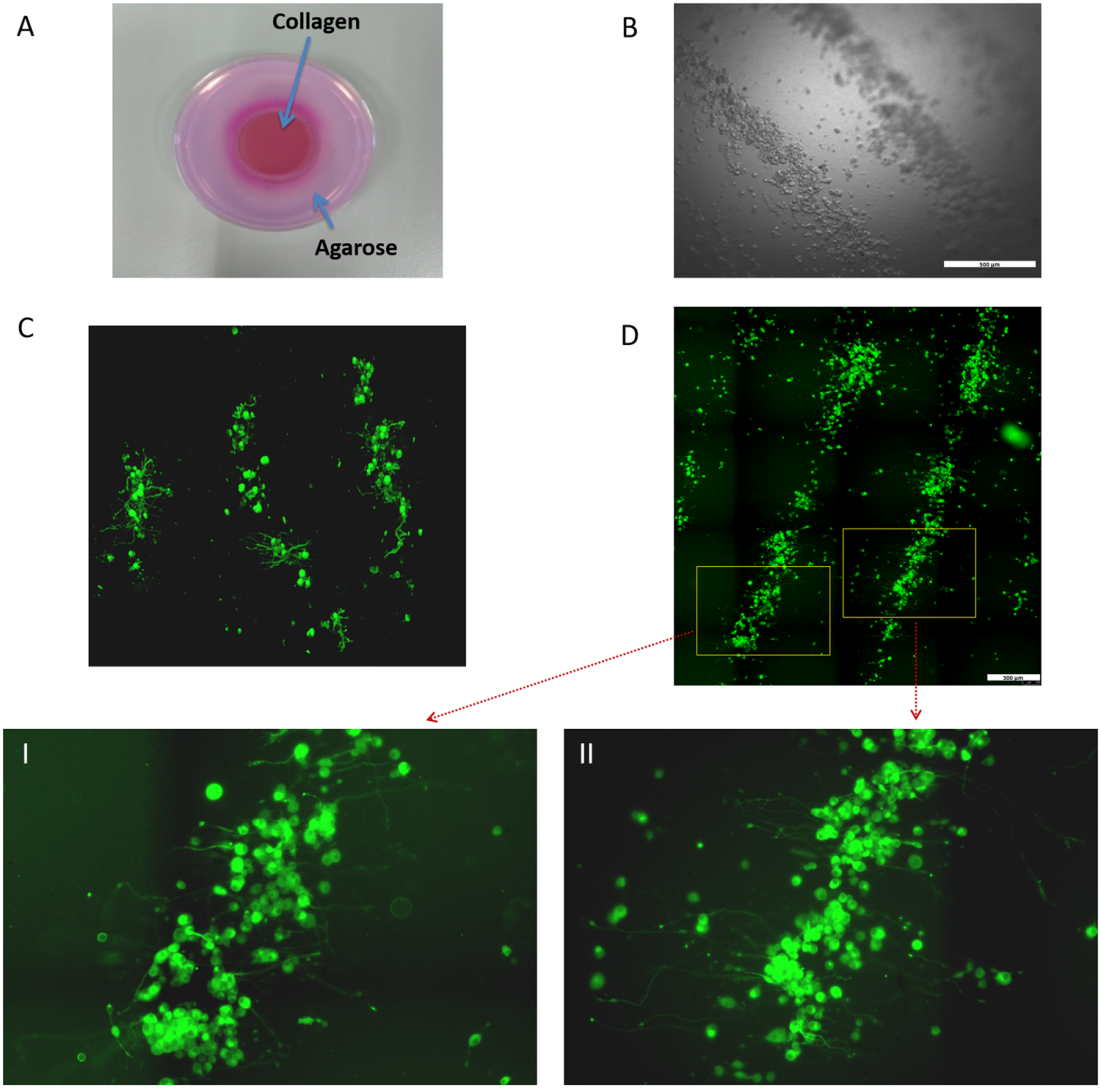
Directed neurite outgrowth of cells on top of a 3D collagen hydrogel. (**A**) Image of the 3D collagen hydrogel after acoustic manipulation of cells, after removing the piezoelectric radial resonator. (**B**) Bright-field microscopic image of DRG neuron arrangement with BAWs on top of the 3D collagen hydrogel. Scale bar = 500 µm. (**C**) Confocal microscopic image demonstrating directed neuronal growth of DRG neurons after two days. (**D**) Fluorescence microscopic images of two adjacent patterned rings from PC12 cells, six days after the BAWs arrangement. Insets I & II show that cells exhibit massive directed growth from one patterned ring to the other. Scale bar = 300 µm.

After demonstrating successful acoustic arrangement of the cells, we examined how this arrangement affects neuronal branching tree growth. We used the BAW setup to arrange primary DRG neurons on top of a solidified 3D collagen hydrogel scaffold (Fig. 4A). The collagen gel solution was poured into the piezoelectric reservoir and allowed to solidify (>45 min) prior to the acoustic manipulation. Then, after the gel solidified, the DRG neurons in the medium were added to the piezoelectric radial resonator and the BAWs were applied. The acoustic waves remained active for 30 min to maintain the cell arrangement until the cells descended onto the solidified gel due to gravitation (Fig. 4B). To trace neuronal growth, neurons were immunostained for neurofilaments (NF) and imaged by confocal microscopy. Figure 4C shows neurite outgrowth of DRG neurons two days after plating. It can be seen that neurons grew their neurites in a relatively directed manner towards neighboring clusters. Z-stack images of the neurons revealed that while growing towards neighboring clusters the branches infiltrated into the gel matrix (Supplementary Video SV2). To evaluate the neurite outgrowth directionality, we arranged and grew differentiated neuron-like PC12 cells on the gel for six days. To image the complete rings with high resolution that would later allow measuring the directionality of the neuronal growth, we imaged small areas at high resolution and then stitched them together (Supplementary Fig. S6). Figure 4D shows extensive neurite outgrowth similar to our previous findings for DRG neurons.

**Figure 5:**
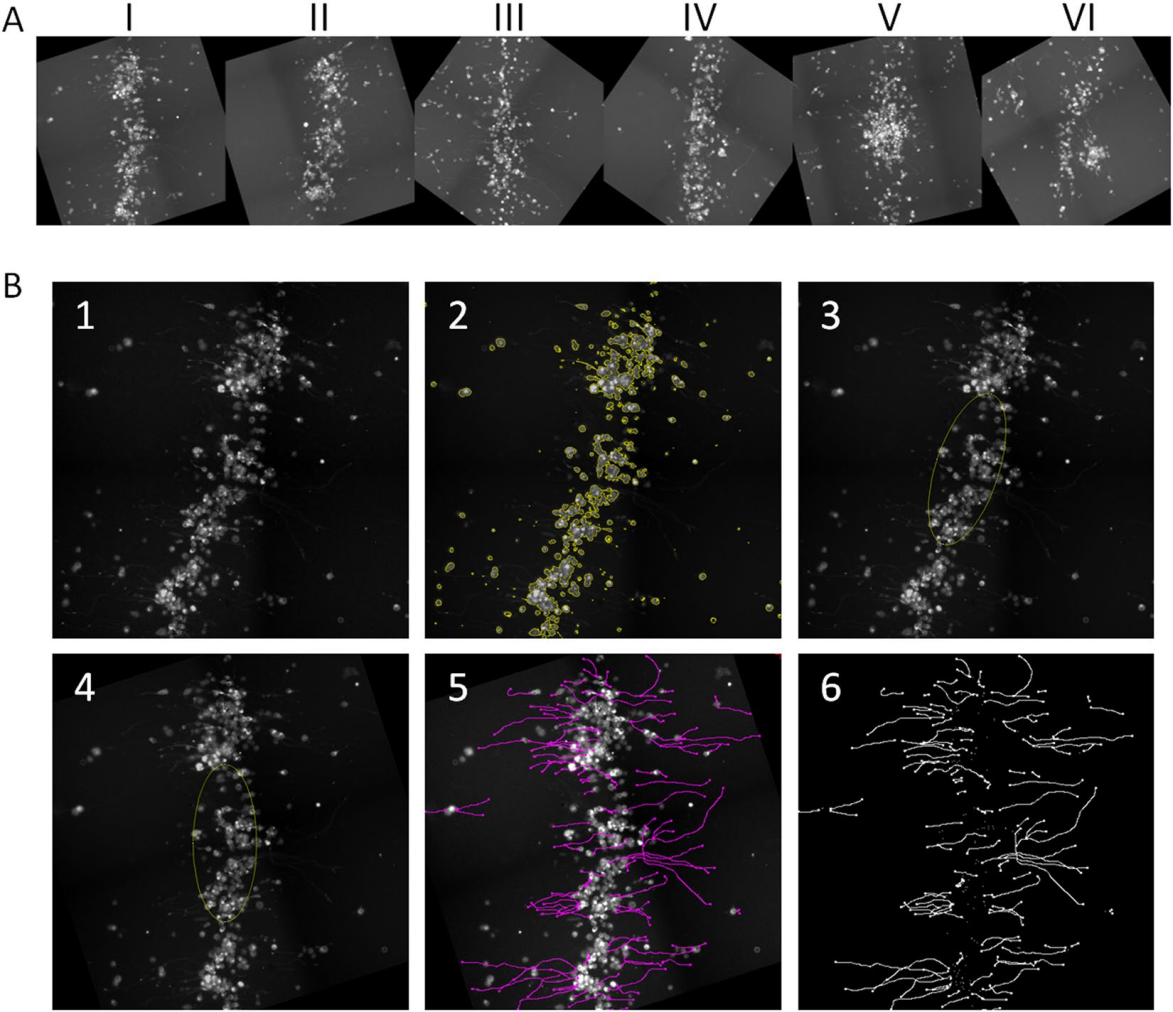
Branches extraction from cell segments created by BAWs for directionality analysis (**A**) Fluorescence micrographs of six different cell segments after alignment. (**B**) Stages of the alignment and tracing processes: (1) the area prior to image processing, (2) an automated selection of body cells (yellow), (3) automated ellipse fitting (yellow), (4) a rotated image by aligning the ellipse to a predetermined orientation, (5) neurite tracing (pink) by using the semi-automated Simple Neurite Tracer plugin, (6) an image of only-labeled neurites.

To quantify the directionality of the branches, we analyzed their orientation at various regions along the concentric rings. We aligned all measured stripes prior to the directionality analysis. Figure 5A shows six independent segments of such an alignment. Figure 5B shows the stages of the alignment and tracing processes that include the following: (1) the examined area prior to image processing, (2) an automated selection of body cells (yellow), (3) automated ellipse fitting (yellow), (4) a rotated image by aligning the ellipse to a predetermined orientation, (5) neurite tracing (pink) using the semi-automated Simple Neurite Tracer plugin, (6) an image of only labeled neurites.

**Figure 6:**
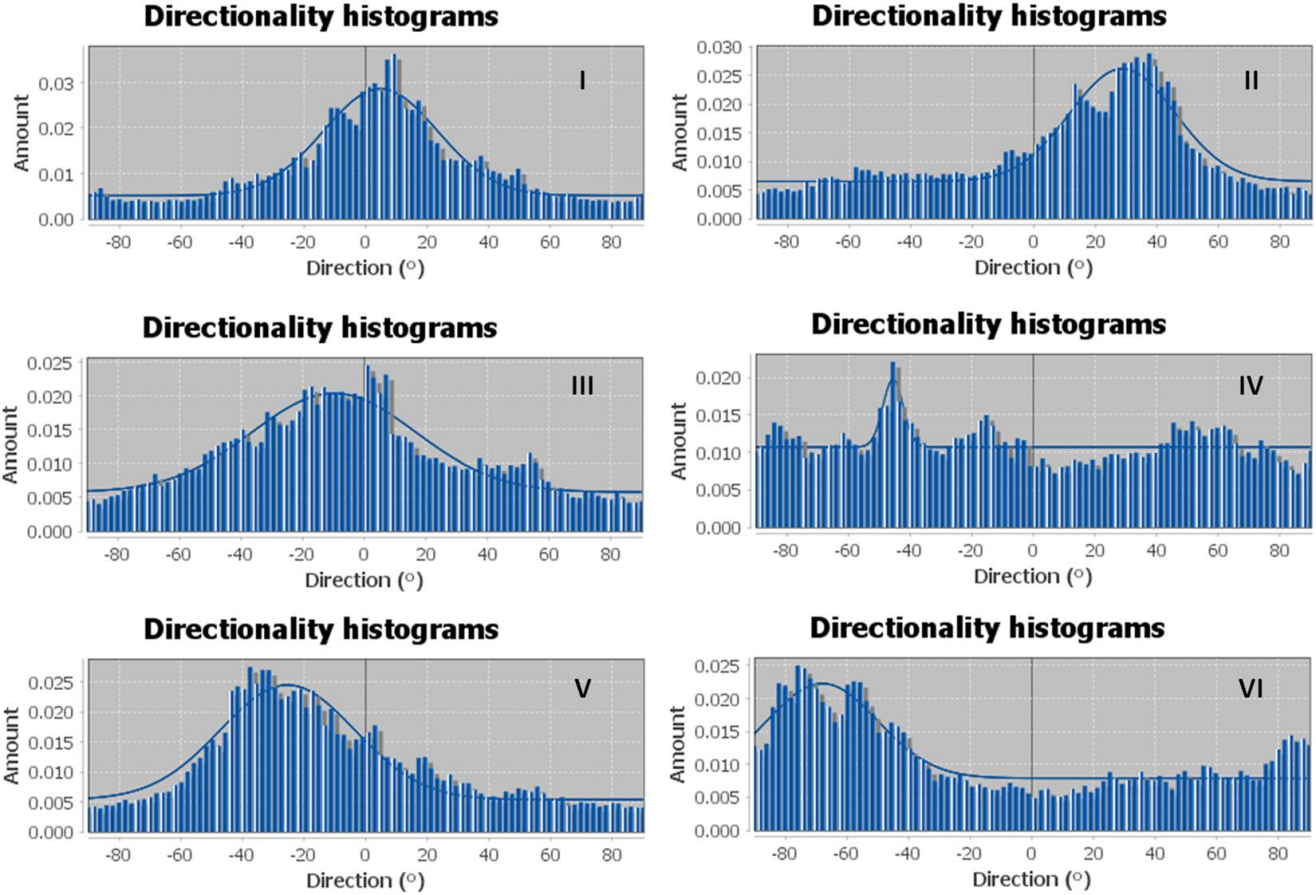
Angular orientation histograms of the BAWs patterned cell segments showing the preferred neurite orientations for five of the six segments.

The processed images revealed that the patterned cells exhibit substantial directed outgrowth. Each processed image was then analyzed for directionality. The directionality histograms (Fig. 6) show a single dominant peak for 5 of the 6 analyzed segments. A peak in the histogram represents a preferred orientation and its position describe the angle of the directionality in respect to the aligned stripe, where 0° is east (perpendicular) and the angles are counterclockwise. It can be seen that the preferred branches outgrowth direction may be different from stripe to stripe. This might be due to the different spatial location of each stripe in the circle and the fact that each stripe may receive different cues from other nearby cell clusters. Since the directionality of neuronal outgrowth is strongly affected by the ability of the growth cone at the tip of the growing process to measure chemical cues^80–82^, our observation suggests that the directionality is governed by chemical signals released by neighboring clusters. Physical environmental cues^60,61,64^ such as the orientation of fibers in the gel, can also affect neuronal outgrowth^62,63^. However, to prevent any directionality of the gel fibers, which may affect the neuronal growth, we applied the BAWs only after the gel had been solidified and we have not found any preferred orientation. Applying the BAWs during the gelation process has led to a swirling vortex-like pattern along the hydrogel fibers (Supplementary Fig. S5) and was therefore avoided.

The ability to pattern neurons on hydrogel matrices at large scales and control the direction of neural outgrowth, without the need for preliminary physical or chemical modifications, points out the potential use of this method to reconstruct specific structures for various uses ranging from basic research to neuronal tissue engineering applications.

## Conclusion

We have used acoustic waves to organize neurons into clusters forming networks without the need for pre-patterned physical or chemical cues. We used two *in vitro* cellular models: (1) PC12 cells, differentiated and non-differentiated, and (2) primary DRG neurons exhibiting high cell viability following the manipulation. By applying two types of acoustic waves, we could organize line clusters and concentric clusters in a highly controlled manner. By changing the acoustic application time, voltage intensity, cell density, and chip structure, we could modify the cluster patterns. Moreover, the cell body’s organization also affected the outgrowth directionality, showing a tendency to elaborate branches to adjacent clusters. In addition to the resulting cell patterns, the two standing SAW and BAW devices are different in their application method and ease of use. Thus, the device of choice should be based on one’s needs and the required scale of organization. For example, if one has to precisely place individual cells on electrical components for brain-machine interface studies then using SAW devices would be the preferred choice. On the other hand, rapidly building artificial constructs of cells with hydrogel that mimic specific brain areas such as the pyramidal layers of CA1 and CA3 in the hippocampus, Purkinje layer in the cerebral cortex, or to create improved tissue engineered grafts to treat peripheral nerve injuries, would require usage of BAW devices. Owing to the remote manner of its acoustic wave application, this method can be potentially used to manipulate cells also *in vivo*, which opens up new possibilities for nervous system therapeutics.

## Methods

### Standing SAW device fabrication and operation

A 128° Y-rotated, X-propagating, single-crystal lithium niobate 3-inch wafer was used as substrate for the standing SAW device. Pairs of interdigital transducers (IDTs) opposite to each other were fabricated on the surface of the LiNbO_3_^83,84^ using standard soft photolithography procedures (see Supplementary Fig. S1). In brief, a layer of photoresist (thickness 1.4 µm, AZ 5214 E, MicroChem) was spin-coated, irradiated by a UV light source through a chrome mask, and subsequently developed with a photoresist developer (AZ 351B, MicroChem). Next, 5 nm chromium (adhesion layer), followed by 100 nm of gold, was evaporated by an electron beam evaporator (Bestec), and a lift-off process was used to remove the photoresist. The center-to-center distance between the electrodes of the IDTs was 103 μm. The LiNbO_3_ wafer was then cut into separate devices that were attached to electric boards. Wires were soldered from the IDTs to BNC connectors. A radio-frequency signal was generated by an arbitrary function generator (Siglent SDG 5162). The selected frequency was found by applying a signal to one IDT while the other was connected to an oscilloscope (SDS1152CML, Siglent). The maximum electrical intensity (indicating the maximal acoustic field) was found to be at 19.4 MHz.

### SAW device and micro-channel integration

Preparing a micro-channel (illustrated in Supplementary Fig. S1) involved using a UV curable biocompatible adhesive (NOA 60, Norland Optical Adhesives) to attach two glass spacers (~100 µm thick) in parallel, to the surface of a glass petri dish (0.17 mm glass thickness, FluoroDish, World Precision Instruments, FL, USA). A glass cover slip (~100 µm) was then adhered on top of the spacers, thus forming a micro-channel. Various micro-channel heights (100–500 µm) were achieved by stacking several spacers. Micro-channels were sterilized by 70% ethanol rinsing followed by 45 min UV light sterilization in a laminar flow hood. The micro-channels were then coated with a type-I collagen to facilitate cell attachment. The micro-channels were placed on top of the standing SAW device with silicon oil (IMMOIL-F30CC, Olympus) used for acoustic coupling. While the SAW devices are reusable, in order to prevent contamination the glass micro-channels are disposable and each micro-channel was used only once.

### Standing BAW preparation and operation

A type-II (lead zirconate titanate, PZT) acoustic radial resonator (inner diameter 22 mm, outer diameter 26 mm, length 20 mm) with a center resonance frequency of 1.1 ± 0.1 MHz was purchased from APC International. To prevent toxicity from the metallic coating of the resonator, it was dip-coated in a Sylgard TM184 silicon elastomer base and curing agent (Dow Corning) mixed at a 10:1 weight ratio. Curing was performed at 80 °C for two hours. The coating (polydimethylsiloxane, PDMS) thickness was found to be ~70 µm. For sterilization, the coated resonator was rinsed with 70% ethanol and placed in a laminar flow hood with UV light for additional 45 min. BAWs experiments conducted inside a sterile CO_2_ incubator. PC12 cells were plated in the resonator and kept in the incubator for 7 days for viability evaluation (Fig. S4). The resonator was driven by a Siglent SDG 5162 function generator. To fixate the resonator, the device was surrounded by an agarose gel (5%, HydraGene) that was poured around it. The BAW devices are reusable and were sterilized between usages.

### Collagen gel preparation

The following mixture was prepared under sterile conditions in a laminar flow hood. A microfuge tube is held on ice during the entire procedure to prevent initiation of gelation. A sterile Collagen (type-I rat tail extract, in 0.02 M acetic acid, an initial concentration of 3.35–4 mg/mL, BD-Bioscience, Bedford, MA, USA) was added to the cold microfuge tube and diluted by addition of 10X DMEM (Sigma Aldrich Co, Stienhiem, Germany) and 7.5% sodium carbonate (both filter sterilized). Phenol red in the DMEM indicates the physiological pH. The collagen concentration is then brought to a final concentration of 3 mg/mL according to the initial collagen batch and the 10X DMEM is then brought to a final concentration of 1X. Gentle pipetting is initiated after adding each material, making sure not to introduce bubbles into the tube. Next, 1 ml from the final gel solution is poured into the piezoelectric tube and is left to solidify for 45 min before initiating the acoustic manipulation.

### Mouse dorsal root ganglia (DRG) neuronal culture

DRG neurons were isolated from 7-week-old C57bl mice. The procedure was conducted in accordance with animal care and protection regulations. Ethical approval was obtained from the University of Bar Ilan Ethics Committee (approval number: 24042015). DRGs were dissected from three mice. Surrounding connective tissues were carefully removed and ganglia were dissociated for 20 min at 37 °C with 1000 U of papain, followed by 20 min in 10 mg/mL collagenase solution, and 12 mg/mL dispase-II at 37 °C. Next, the ganglia were triturated in HBSS, 10 mM Glucose, and 5 mM HEPES (pH 7.35) by repeated passage through a constricted Pasteur pipette. Neurons were recovered through 20% percoll in L-15 medium. Neurons were counted and 5 × 10^5^ cells were suspended in 1 ml of F-12 antibiotic-free medium, supplemented with 10% FBS, and were loaded on top of the solidified collagen gel for BAW manipulation. Neuronal cultures were developed for two days, and then cultures were fixed and stained.

### PC12 neuron-like cell culture

PC12 cells were grown in suspension in a RPMI medium supplemented with 10% horse serum (HS), 5% fetal bovine serum (FBS), 1% L-glutamine, 1% penicillin-streptomycin, and 0.2% amphotericin, in a humidified incubator at 37 °C containing 5% CO_2_. For cell patterning, the cells were suspended in serum-reduced media (1% HS). For the standing SAWs manipulation, up to 30 µl of cell-containing medium (1 × 10^4^ to 12 × 10^4^ cells/100 µl) were loaded into the channel that was built on top of a collagen type l-coated glass-bottomed petri dish. For the BAWs manipulation, 1 ml of cell-containing medium (1 × 10^5^ to 1 × 10^6^ cells/ml) was loaded on top of the solid collagen gel. To induce PC12 differentiation, 24 hr after cell patterning Murine β-NGF (Peprotech, Israel) was added to the medium at a final concentration of 50 ng/mL. Every two days, fresh medium containing Murine β-NGF was added to the cells.

### Immunofluorescence staining

The medium was removed from the *in-vitro*-cultured collagen gels and each gel culture was fixed with 4% paraformaldehyde for 15 min at room temperature (RT). Next, the gel cultures were washed with phosphate-buffered saline (PBS) and permeabilized with 0.5% Triton X-100 in PBS (PBT) for 10 min. Then, cells were incubated in a blocking solution (containing 1% BSA and 1% NGS in 0.2% PBT) for 45 min at RT. Next, DRG neurons were incubated with mouse anti-neurofilament H (NF-H) for 1.5 hr at RT, and PC12 cells were incubated with a rabbit antibody to α-tubulin (Santa Cruz Biotechnology, Inc., Santa Cruz, CA) for 1.5 hr at RT. The cultures were rinsed 3 times with PBS and incubated for 45 min at RT with Alexa Fluor 488 conjugated Donkey anti-mouse IgG H&L for DRG neurons, or with Cy2-conjugated AffiniPure Donkey Anti-Rabbit secondary antibody (Jackson ImmunoResearch Laboratories, Inc., West Grove, PA) for PC12 cells. Following incubation, the cultures were rinsed with PBS. Fluorescence images were acquired by using either a Leica SP8 scanning confocal microscope, driven by LasX software, or Leica DMI8 wide-field microscope. Images from the wide-field microscope were tiled and stitched using the LasX acquisition software.

### Analysis

Neurite directionality was analyzed using Fiji - ImageJ software. First, images containing independent stripes for analysis were randomly selected from different rings. Each image captures an area of 1.5 × 1.5 cm, and contained only one continuous defined stripe. To semi-automatically select the patterned cell bodies, we used the ‘Versatile Wand Tool’ plugin. Following selection, we automatically fit ellipses. The major axes of the ellipses were then rotated to the same orientation. Next, by using the ‘Simple Neurite Tracer’ plugin, we semi-automatically traced all the neurites that projected from the cell bodies. Then, we used a thresholding technique to separate the labeled neurites from the rest of the image. For neurite directionality analysis, we used the ‘Directionality’ plugin. Angles are reported in their common mathematical way. 0° is the east direction and the orientation is counterclockwise.

## Supplementary information

Supplementary material.
