# Supplementary material for: Large-scale acoustic-driven neuronal patterning and directed outgrowth
Source: Sci Rep. 2020 Mar 18;10:4932. doi: 10.1038/s41598-020-60748-2 (PMC7080736; doi:10.1038/s41598-020-60748-2)
Supplement: Supplementary file 1 — Supplementary material. [file 41598_2020_60748_MOESM1_ESM.docx]

**Supplementary Information**

**Large-scale acoustic-driven neuronal patterning and directed outgrowth**

*Sharon Cohen, Haim Sazan, Avraham Kenigsberg, Hadas Schori, Silvia Piperno, Hagay Shpaisman^*^ and Orit Shefi^*^*

**
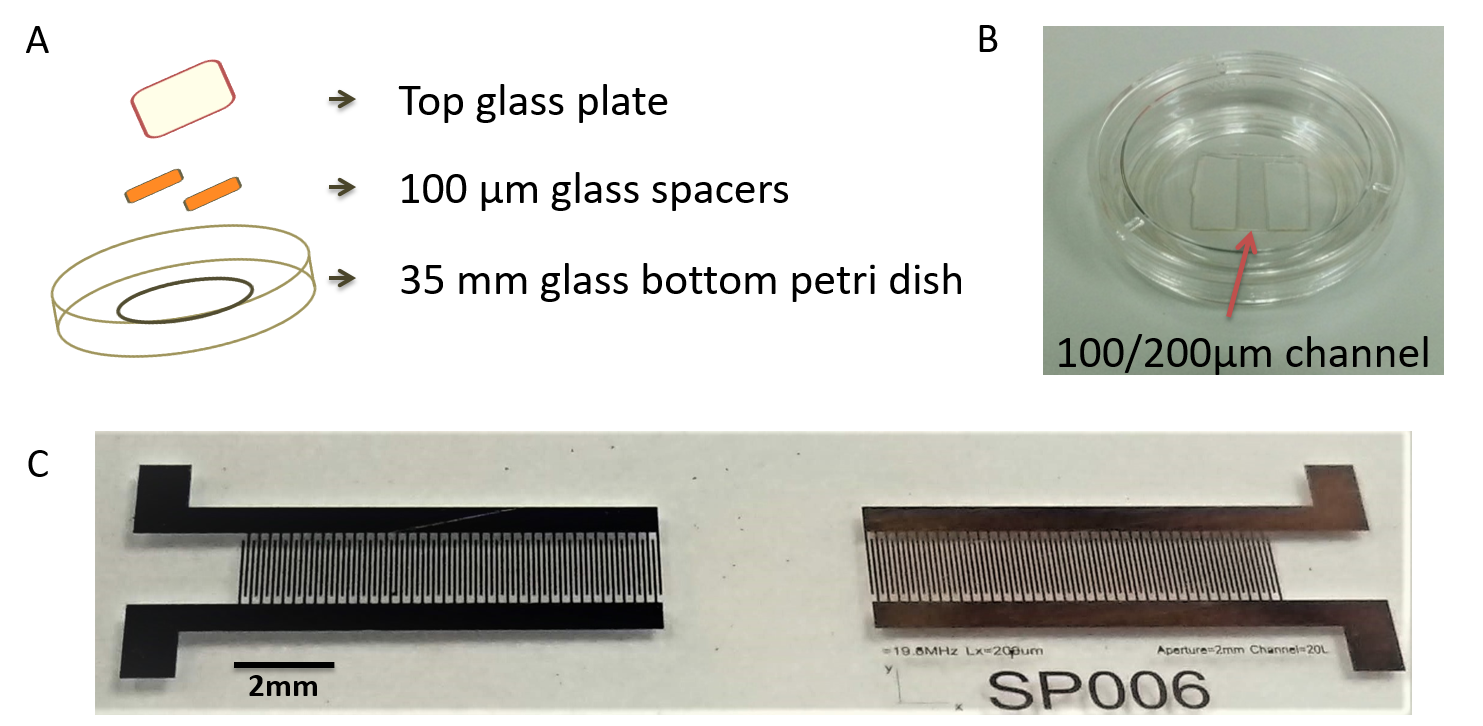
**

**Supplementary Figure S1.** A) Schematic diagram of the reaction channel components, built on top of a glass-bottom petri dish. Glass spacers were glued on both sides of the petri dish and covered with a glass cover slip to form the channel. B) Photograph showing the channel design. The medium with cells was placed at the channel’s edge (red arrow) and filled the channel. C) Image of the IDTs fabricated on a piezoelectric LiNbO_3_ substrate.


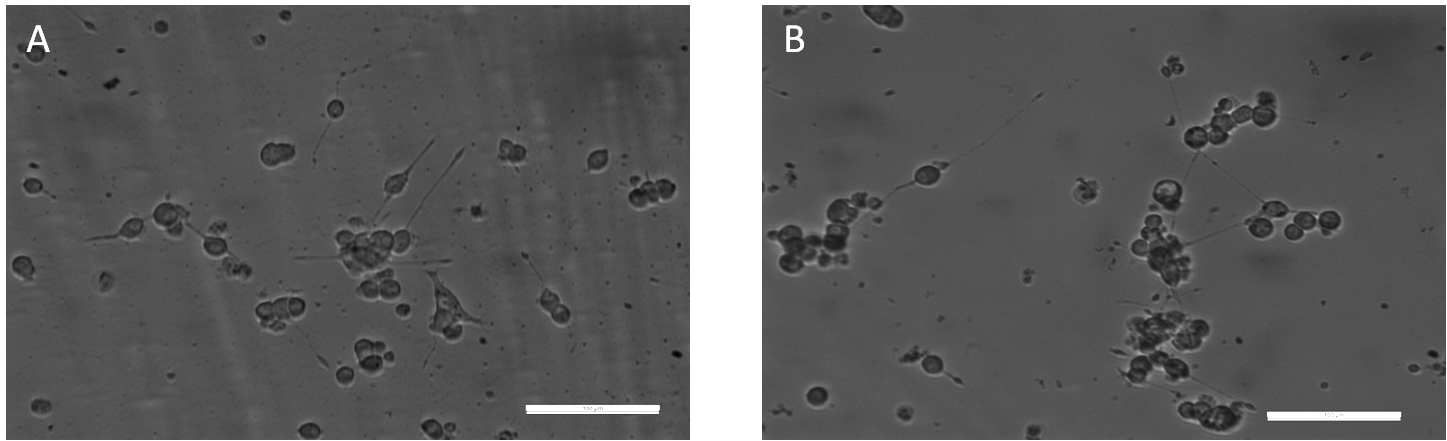


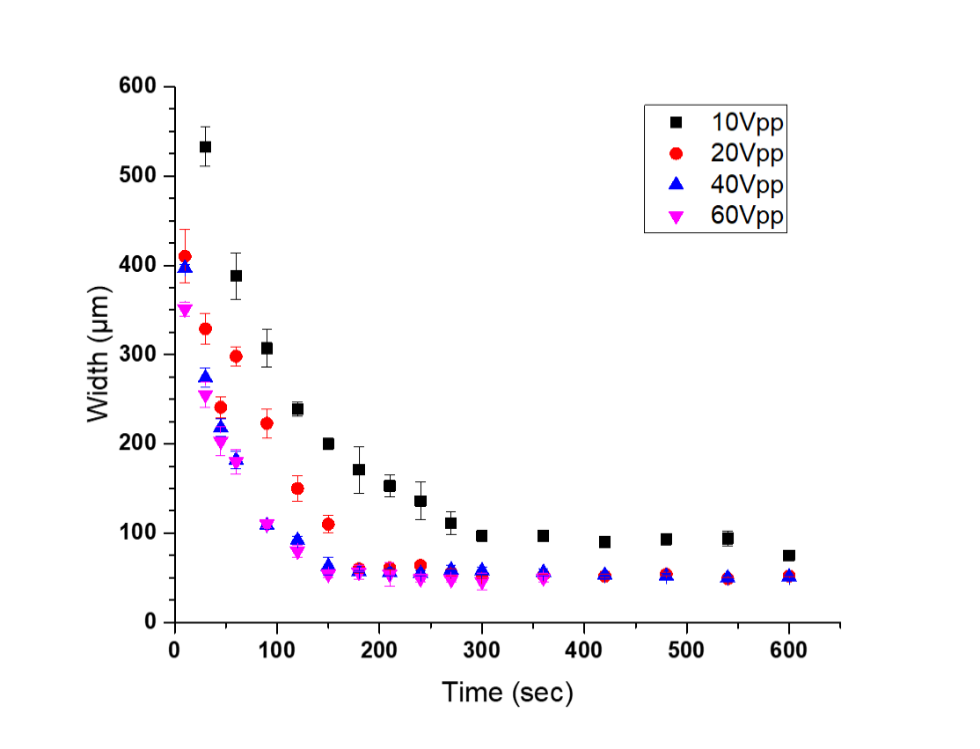
**Supplementary Figure S2.** PC12 cell neurite outgrowth following SAW manipulation. A normal pattern of neurite outgrowth was observed both without SAWs (A) and with SAWs (B), indicating that the SAWs were not harmful to the cells. Scale bar = 100 µm.

**Supplementary Figure S3.** Effect of different voltage intensities of BAWs on 2µm polystyrene (PS) beads arrangement. Width (of the second ring from the center) as a function of time for various applied voltages using 2µm PS beads. Each data point represents an average of ten individual measurements. Due to the relatively high concentration of PS beads (compared to concentration of cells) before applying the BAWs the microscope’s field of view is covered with PS beads. When BAWs are applied, the PS beads become more concentrated (as rings) and therefore the graph shows a decrease in width over time while cells with relatively low concentration seem to be growing over time (see Figure 2D).


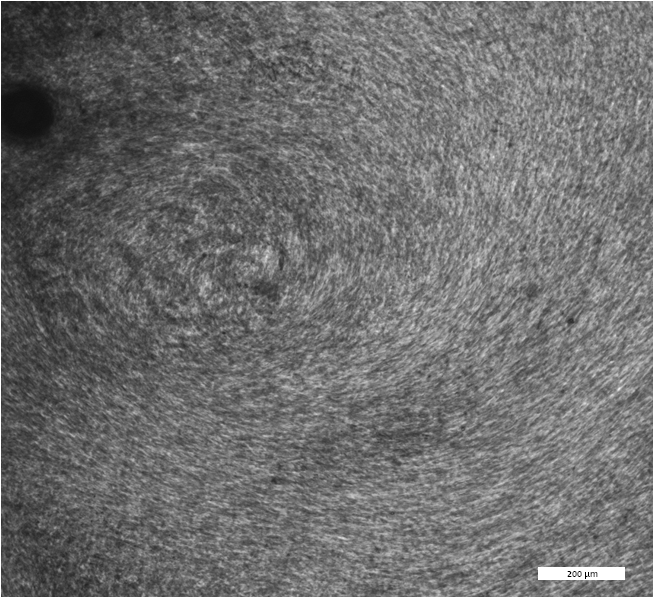

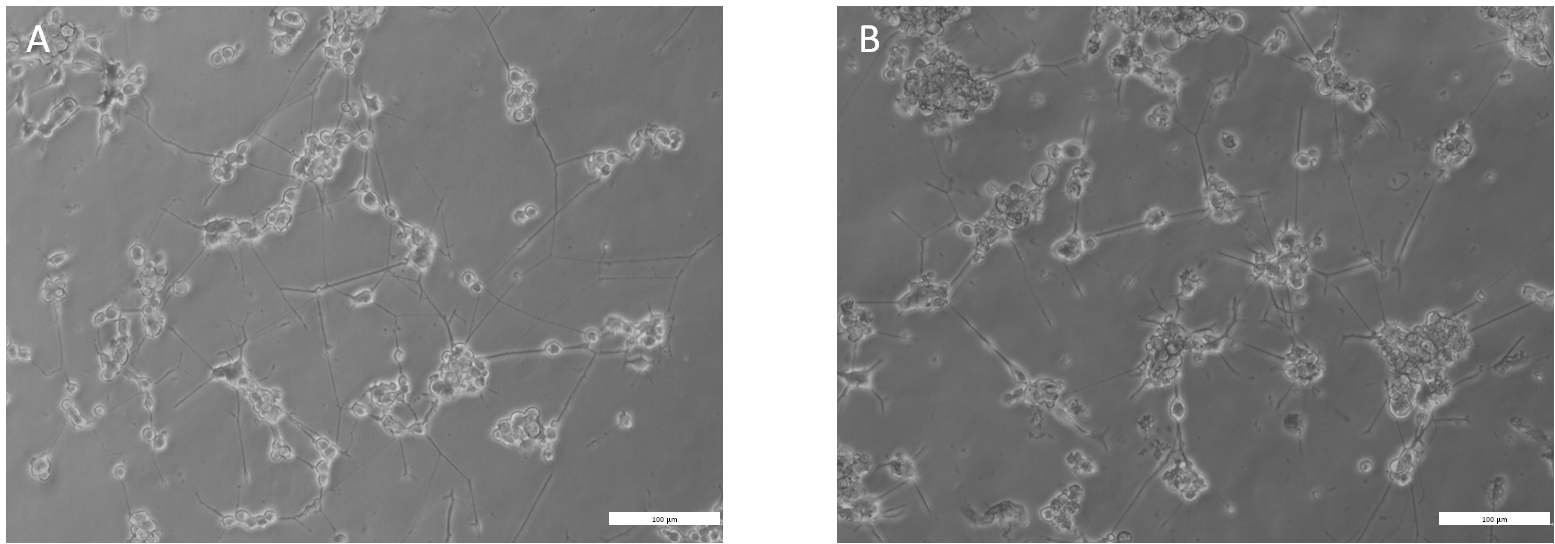
**Supplementary Figure S4.** PC12 cell adhesion and growth inside the PDMS-coated piezoelectric tube. A week after plating, PC12 cells were attached to the substrate, extended neurites, and formed networks both in the control (A) and inside the piezoelectric tube (B). This observation indicates that the PDMS-coated piezoelectric tube was not toxic to the cells. Scale bar = 100 µm.

**Supplementary Figure S5.** Bright-field microscopic image of 3mg/ml collagen hydrogel solidified under the influence of BAWs at 25°C. Gel fibers demonstrate the arrangement in a swirling vortex pattern. Scale bar = 200µm.

**
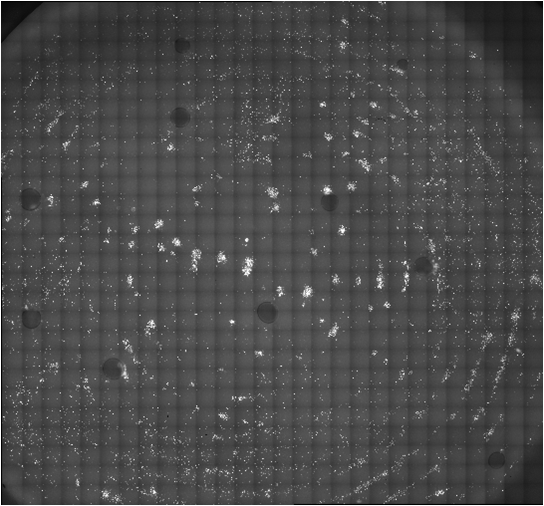
**

**Supplementary Figure S6.** A 28x26 tiled and stitched image of PC12 cells on top of a 3D collagen hydrogel shows the rings that were generated following acoustic manipulation. Each square has a size of 550x550µm.

**Supplementary Video SV1** Assembly process by standing SAWs of PC12 cells in a 200µm high channel with acoustic waves generated at 19.4 MHz and 10Vpp.

**Supplementary Video SV2** Primary DRG neurons growing on top of a 3D collagen hydrogel scaffold after standing BAWs arrangement. Confocal Z-stack images demonstrate branches infiltration into the gel matrix.
